# Televisit with TytoHome™ device in medically complex child in long-term mechanical ventilation: a pilot study

**DOI:** 10.1186/s13052-025-01885-0

**Published:** 2025-02-12

**Authors:** Alessandro Onofri, Nicola Ullmann, Elisabetta Verrillo, Maria Giovanna Paglietti, Martino Pavone, Renato Cutrera

**Affiliations:** Pediatric Pulmonology & Cystic Fibrosis unit - Bambino Gesù Children’s Hospital, IRCCS, Rome, Italy

## Abstract

**Background:**

During the pandemic, the pneumology team at Bambino Gesù Children’s Hospital highlighted that telemedicine was a valuable tool for remotely managing the medical needs of children with medical complexity (CMC). Following the telemedicine experience during the emergency phase, a telemedicine service was established, and new tools were tested to optimize televisits and the overall eHealth approach for patients. In this context, the TytoHome™ device was tested for performing objective examinations remotely. This pilot study, conducted at our hospital, explored the management of CMC patients on long-term mechanical ventilation via the telemedicine platform and the TytoHome™ device.

**Methods:**

This study involved the treatment of 10 pediatric patients over one year using this approach. The patients were already receiving care at our hospital and were undergoing long-term mechanical ventilation (LTV) at home—4 on invasive mechanical ventilation (IMV) and 6 on non-invasive ventilation (NIV). A database was developed to collect patient data, including personal details, vital parameters, objective examinations, audio quality, and patient satisfaction. A descriptive analysis was subsequently performed using the data collected during the earlier stages of the study.

**Results:**

The utility of the TytoCare device for medically complex children was evaluated. The families were “satisfied” with the remote follow-up visits, and healthcare personnel rated the audio quality of the visits as “good.”

**Conclusions:**

In conclusion, the remote management of these patients using the Tyto device offered several advantages. In our experience, Tyto proved to be a useful tool for the remote medical management of complex patients.

## Introduction

Telehealth has been expanding worldwide and has proven to be an effective way to deliver healthcare [1, 2].

E-Health is defined as “the cost-effective and secure use of information and communication technologies to support health and health-related fields, including healthcare services, health surveillance, health literature, health education, knowledge, and research.” Although e-health encompasses a variety of activities, telemedicine and mobile health (m-health) are the most commonly used in everyday clinical practice [3].

Telemedicine is a term coined in the 1970s, which literally means “healing at a distance.” Since 2007, the World Health Organization (WHO) has defined telemedicine as the provision of healthcare services at a distance using information and communication technologies (ICT) by healthcare professionals [4]. Since 1998, Patch et al. [5] have compared video-conferencing examinations with traditional face-to-face methods. They concluded that the evaluation of patients via telemedicine was as effective as in-person consultations.

The recent COVID-19 pandemic accelerated this digitalization process [6]. During the pandemic, telemedicine was primarily applied in the homecare setting to screen high-risk and low-risk COVID-19 patients [7].

Children with medical complexities (CMC) experience medical fragility and intensive care needs, as they often have congenital or acquired multisystem diseases and technological dependencies for activities of daily living [8]. During the COVID-19 pandemic, these children experienced disruptions in homecare services. Therefore, a virtual space was required to conduct visits and consultations, avoiding the risks associated with hospital visits [9].

In response, our department established a virtual care service for the pulmonary care of CMC. We used telemedicine to support patients and caregivers, developing strategies to follow up with these patients remotely. We conducted televisits using dedicated platforms and remote monitoring of home ventilators to assess clinical conditions, ventilator issues, and disruptions in home-care assistance. We found that the use of telemedicine for CMC patients was feasible and helped to minimize in-person visits [10].

In this context, we had the opportunity to trial a device for remote objective examinations shortly after the pandemic: TytoHome™. Specifically, it allows for objective examinations of the chest (heart tones and lung sounds), otoscopic, dermatological, and oral examinations, as well as the detection of vital parameters (heart rate and body temperature). The device consists of a touch-sensitive screen with an integrated high-resolution camera and an infrared forehead thermometer. It can be paired with digital stethoscopes, otoscopes, or tongue depressors. The device is connected to a mobile phone or tablet to enable video conferencing while capturing data from physical examinations [11]. The dedicated platform successfully met expectations for live teleconsultations and exam records.

### Aim of the study

At the Bambino Gesù Children’s Hospital Department of Pneumology and Cystic Fibrosis in Rome, we conducted a pilot study investigating the home management of children with CMC on long-term ventilation (LTV) through the use of TytoHome™ and its web platform.

The primary aim of the study was to assess the efficacy of a telemonitoring program using the TytoHome™ device and to evaluate the usefulness of the platform and the devices connected to it. The secondary objective was to determine the effectiveness of using the device to conduct televisits and perform remote medical objective examinations for children with medical complexity. We compared the data collected during the study period with a set of retrospective data from the same patients gathered during the year prior to the start of the study.

## Methods

The pilot study conducted at our hospital lasted 12 months and involved a total of 10 patients.

The patients included in the study were selected based on the following criteria: under 18 years old, already under follow-up at our hospital, and receiving long-term home ventilation treatment. Additional inclusion criteria were: a caregiver/patient with good IT skills (if collaborative), and access to an adequate electronic interface (smartphone and Wi-Fi connection). The telemedicine team, consisting of a pneumologist, nurse, and respiratory physiotherapist, conducted televisits every 2 months, with additional visits as needed.

Notably, during the study year, the patients continued their regular follow-up program.

### Training to the physician

The pre-recruitment phase involved training to teach patients and caregivers how to properly use the device and interpret lung sounds with the TytoHome™ system. Specifically, the physician of the team conducted concurrent objective examinations using both a stethoscope and the TytoHome™ device, examining both healthy subjects and those affected by various chronic lung diseases (such as cystic fibrosis, bronchiectasis, etc.). Additionally, the healthcare professionals involved were trained on how to effectively use the web platform. TytoCare’s technical support team assisted users in employing both the device and the web platform.

### Enrollment and training to the patients

The project was offered to patients who met the inclusion criteria, and an Informed Consent Form (ICF) was signed at the time of enrollment. The first educational training session was provided to patients (if collaborative) and their caregivers through a video call by TytoCare’s technical service. The necessary devices were loaned to the families and delivered to their homes after the ICF was signed.

Notably, televisits using TytoHome™ were conducted in addition to standard care. Therefore, the follow-up program for each patient remained unchanged during the study.

### Children with medical complexity (CMC)

The population in our study includes CMC patients who are managing multiple severe medical conditions. Rogers et al. proposed that CMC patients should be defined by four common parameters: complex chronic conditions, advanced medical technology, functional limitations, and care coordination across the healthcare continuum [12].

The recruited patient sample is followed up through personalized coordination of healthcare services and resources, aimed at ensuring effective care for this patient group. This coordination may involve collaboration among physicians, nurses, physiotherapists, and other health professionals. In most cases, home care services and high-tech devices are planned to prevent disease progression and improve the quality of life for both patients and caregivers in their daily activities.

### TytoHome™ device

TytoHome™ is a small, innovative device designed for remote objective examinations. This tool includes an electronic microphone for performing thoracic exams and a high-resolution camera for various assessments, such as dermatological examinations. It also features a stethoscope for heart and lung auscultation, a digital otoscope for visualizing the tympanic membrane, a digital thermometer, a digital oximeter, and a tongue depressor [13]. The transmitted data and images are recorded and can be sent to the clinician at any time. Additionally, a video conference mode is available for conducting multidisciplinary meetings. The device is paired with a mobile application for families, enabling synchronous or asynchronous telemedicine visits, recording vital signs, and compiling a patient symptom diary.

### Televisit

Televisits are conducted using the TytoHome™ system, and its digital platform is designed for remote physical examinations.

In our experience, the televisits were carried out on the TytoHome™ online platform and proceeded in two steps: first, an anamnestic evaluation, followed by a pulmonary examination.

The anamnestic evaluation was standardized using common criteria for all patients involved in the project. It initially assessed the baseline clinical situation, the treatment and home care plan, and any remote interventions, all of which were reviewed during each televisit. Following this, the current pharmacological therapy, recent exams, and ventilation parameters were evaluated. Information regarding clinical stability, the accuracy of home care services, and any ventilatory issues was also collected.

Next, the objective examination was performed. The primary focus of the examination was the evaluation of chest sounds and the collection of vital parameters. A focused assessment was conducted using the optional Tyto tools (video camera, tongue depressor, otoscope, etc.) as needed.

Two methods were used to perform the examination: a live examination or a recorded exam.

The live examination was guided by the physician and performed by the patient’s caregiver. If a recorded exam was chosen, the procedure was agreed upon with the team and involved the caregiver performing the exam, which was then recorded and sent to the platform at a later stage.

### Analysis

A specific database was created to collect the following data: general features, vital parameters, objective examination results, number of respiratory exacerbations (defined as requiring intervention and modification of the patient’s usual care plan), number of interventions made by the team based on the televisit and TytoHome™ objective exams, most frequently recorded information (lung sounds, heart sounds, otoscopy, pharyngeal evaluation), quality of images and sounds as reported by the physician, patient and family satisfaction, number of hospital visits via the Emergency Department, and number of scheduled hospitalizations.

Patient and family satisfaction were assessed through an oral questionnaire. The questions focused on satisfaction with individual visits, and responses were assigned a numerical score from 0 to 3 (0 = unsatisfied, 3 = completely satisfied). The quality of images and sounds reported by the physician was evaluated at the time of the visit and assigned a numerical score from 0 to 3 (0 = insufficient quality of data, 3 = high quality of data).

Additionally, the aforementioned data was supplemented by a set of retrospective data on each patient’s clinical condition and hospital access one year after the study began. This retrospective data included: number of hospital visits via the Emergency Department (ED), number of scheduled hospitalizations and outpatient treatments, and number of respiratory exacerbations.

The data was collected in a database, and statistical analysis was performed using Excel (version 16.0). A descriptive analysis was carried out to extrapolate results, utilizing sample distribution, absolute frequencies, percentages, and central tendency indices, such as the mean and standard deviation (SD), for quantitative variables. A 5-point Likert scale was used to measure satisfaction.

## Results

The pilot study conducted at our hospital involved a total of 10 patients with medical complexity, with a mean age of 13 years (SD ± 4.5), of whom 5 (50%) were male. All patients were already receiving treatment at our hospital and had previously undergone long-term home ventilatory treatment (4 patients on invasive mechanical ventilation and 6 patients on non-invasive ventilation).

The main characteristics of the patients are summarized in Table 1, and the data on the patients’ medical devices and home assistance are presented in Table 2.

**Table 1:** Main patients’ characteristics

Patients’ characteristics	
*Age*,* mean (SD)*	13 (± 4.5)
*Sex*,* n*	
Male	5
Female	5
*Diagnosis*,* n*	
CP	3
NMD	5
Other	2
*Ventilation Mode*,* n*	
NIV	6
IMV	4
*Vital signs*,* median (SD)*	
SpO2 (%)	96 (± 2)
FC (bpm)	82 (± 17)

Note: Abbreviations (CP: cerebral palsy; NMD: neuromuscular diseases; NIV: noninvasive ventilation, IMV: invasive mechanical ventilation)

**Table 2:** Patients’ devices needed and home care assistance

Patients’ devices and assistance	
*Devices and needs*,* n*	
PEG	4
Tracheostomy	4
Oxymeter	7
Suction machine	5
Cough machine	3
Enteral feeding pump	3
*Assistance at home*,* n (%)*	
Physician	1
Nurse	4
Physioterapist	7

*Note: Abbreviations (PEG*,* percutaneous endoscopic gastrostomy)*

A total of 70 visits were performed from 12th June 2022 to 12th June 2023. 46/70 (66%) of the visits were performed synchronously in the online platform and 24/70 (34%) with the registration of the exam.

The vital parameters were stable across all televisits (Table 1).

Regarding the totalnumber of the synchronous televisits, 30/46 (65%) of them wereperformedthrough live clinical examinations, whilein 16/46 (35%) of visits, the physical examination was not performed.

Specifically, the objective examination wasn’t performed due connectivity issues in 12/16 (75%) visits and due to managing difficulties of the families in 4/16 (25%) visits.

Moreover, 4 cases resulted inconclusive, due to issues with audio tools, counting 30 live examinations performed.

Clinicalexamswere stable in 74% of the visits, altered in 26%. The instrument used was stethoscope in 93% of the exams, 4% otoscope, 4% high-resolution camera. We considered the examination abnormal when pathological lung sounds were identified. In our sample, we primarily observed bilateral medium and coarse crackles (7/8 cases), with crepitant crackles at the right base in one case. All patients with abnormal findings on physical examination were referred for an urgent consultation. In all patients, the physical examination findings were confirmed during the in-person consultation. During the televisits, healthcare professionals were able to evaluate each patient and stepped in to solve problems and needs which emerged during the visit. The team intervened in different ways including hospitalization planning, request for technical assistance, clinical indications, instrumental exams, etc. A total of 17 interventions have been successful, and are listed in Table 3.

**Table 3:** Remote interventions after televisits

Remote interventions	*n* (%)
*Technical assistance*	3
*Hospitalization planning*,* visits*,* sleep studies*	4
*Indications to pharmacotherapy*	4
*Indications for ventilatory/oxygen therapy*	2
*Educational intervention*	4

In the selected pool of subjects, statistically significant differences between the year of analysis and the previous year were not found in terms of: number of planned hospitalization, number of ED access, number of exacerbations.

## Discussion

The primary objectives were to evaluate the performance of the TytoHome™ instrument for conducting objective examinations in CMC patients with LTV and to establish operational procedures for managing these patients at home. Procedures, interventions, and data were collected during televisits to assess the feasibility of this telemedicine program and evaluate the TytoHome™ device for a remote approach to patient care, ensuring safety and effectiveness.

CMC patients have multiple medical comorbidities, increased healthcare needs, and require support from various healthcare professionals [8]. These patients exhibit a range of pathologies, but share a condition of fragility, which leads to high technology use, management of advanced devices, intensive assistance, home care needs, and integration of services [14], as seen in the small sample included in this pilot study (details in Table 2).

There are different models for managing medically complex children, broadly categorized into three types: primary care-centered, consultative or multidisciplinary, and episode-based care. These models reflect the lack of consensus on the best practices for the CMC population [15]. Telemedicine is emerging as a practice, particularly in the primary care-centered model [16].

As early as 1997, Miyasaka et al. defined telemedicine as a practical and effective tool for managing ventilated patients [16]. In the last decade, literature has increasingly focused on the use of telemedicine in CMC patients, alongside the development of new technologies, infrastructures, and devices. Numerous studies confirm the efficacy of telemedicine programs for medically complex children, facilitating early diagnosis, treatment, and support in patient management [17, 18]. Additionally, telemedicine has been associated with reduced healthcare costs [19] and improved parent satisfaction [20], findings consistent with our study.

After the COVID-19 pandemic, telemedicine evolved rapidly [9]. The growing development of telemedicine-related technologies has highlighted the need for a roadmap in setting up telemedicine programs and services. Emerging statements in the literature emphasize the opportunities digital technologies offer for patient management [21–23].

In this context, our project focused on the TytoHome™ device, a tool for remote integrated objective examination. Through our experience, we aim to illustrate the operational capabilities of the tool and the strengths and limitations of a telemedicine program that includes objective examination for CMC patients. We found that TytoHome™’s back office was easy to use, affordable for clinicians, and facilitated the planning and management of telemedicine visits and objective examinations.

We performed 70 visits, both synchronously and asynchronously. The objective examination was within normal parameters in 74% of the cases. Despite the device’s ease of use, 35% of synchronous visits had unassessable objective examinations due to connectivity issues or family management problems. Additionally, 4/30 synchronous visits were unassessable due to issues with the audio tools. These limitations skewed the results, providing only a partial view of the instrument’s effectiveness.

The most commonly used TytoHome™ tool during the study was the stethoscope (93% of visits), with significantly lower usage of other accessories. This likely occurred because, despite the patients’ complexity, the purpose of the visit was primarily a pulmonary objective examination. The global approach to each patient was ensured by specialists on-site and follow-up visits as part of the usual care plan. Other tools were used only when specific issues were reported by caregivers.

The research team emphasized the importance of interacting with patients and parents during remote physical examinations and video visits. These interactions allowed the team to address problems, guide families, and customize care plans to meet patients’ needs. The team concluded that a standardized anamnestic evaluation of domiciliary care could be a useful qualitative tool to assess a patient’s condition before the objective examination, helping identify potential needs. This approach has not been extensively discussed in the existing literature.

The main needs identified during the visits in our study included healthcare planning, sleep studies, pharmacotherapy recommendations, and educational interventions on ventilatory treatment (4 cases). Technical support was necessary in 3 cases, and sufficient assistance was received. Some minor ventilator modifications were required in two cases, due to humidification issues.

The use of the Tyto device in pediatric patients has been discussed in existing literature. Wagner et al. [11] found satisfactory concordance between remote and in-person physical examinations for otoscopy, throat and oral examination, skin examination, and heart and lung auscultation, with some limitations in heart and lung auscultation in infants and abdominal auscultation in children of all ages. The use of the Tyto device resulted in lower diagnostic failure rates and high intra- and inter-reliability for examining the heart, lungs, and ears in pediatric patients with various pathologies [24].

Regarding the use of TytoHome™ in CMC ventilated patients, a study by Notario et al. showed promising results for evaluating objective examination, with satisfaction from both caregivers and clinicians [25]. Despite the small sample size and short observational period (4 months), the authors demonstrated that in-home telehealth devices are feasible and may reduce hospitalizations compared to usual care.

Our study aimed to provide a longer-term evaluation, analyzing 12 months of added telemedicine visits to the usual care treatment and comparing them with retrospective data from the previous year. Similar to Notario et al. [25], the feasibility of using TytoHome™ was appreciated by the feedback from the team (audio quality rated 2/3 on the scale) and caregiver satisfaction (also rated 2/3). Comparing the data from the study year with the previous year, no significant differences were found between the average number of planned hospitalizations (from 1.3 to 1.0) and Emergency Department (ED) visits. However, the number of respiratory exacerbations increased (from 0.2 to 0.9), which could be attributed to higher detection potential. The increased follow-up frequency during the study likely led to the detection of more respiratory exacerbations.

In contrast to Notario et al. [25], our study found a reduction in hospitalizations when compared to the pre-pandemic period (2018), where the median hospitalization rate was twice per year [26]. The data from our study, conducted post-pandemic, indicated an average hospitalization rate of less than two per year. This decrease may be attributed to the pandemic experience, which provided tools for more efficient home management. Furthermore, the additional decrease between the retrospective and study years, when a structured telemedicine program was implemented, supports this hypothesis.

Our study has both strengths and weaknesses. A key strength is the ability to conduct objective examinations remotely, reaching patients in their home setting who would otherwise be difficult to supervise. Additionally, the telemedicine platform enabled information exchange among physicians and professionals in the community.

However, the study also had limitations, including technical issues encountered during the tele-visit. The failure to execute physical examinations in all visits resulted from various problems, including audio and organizational issues with parents, which posed a limitation to the study and prevented the evaluation of all exams. Another limitation was the hospital firewall, which required the use of an alternative internet connection compared to those available within the hospital. Additionally, ventilator data was only collected for patients with ventilators capable of transmitting data, which led to the exclusion of heterogeneous data from our analysis.

## Conclusions

During the study year, the telemedicine team identified both strengths and weaknesses regarding the Tytocare tool and its web platform.

At the end of the visits performed by the team, several advantages of Tytocare in the remote management of medically complex children were noted, such as the prevention of exacerbation episodes and the facilitation of cooperation and communication with healthcare professionals managing the patients at home.

Thanks to the high satisfaction expressed by the families and the excellent audio quality noted by the healthcare team, it can be confirmed that Tytocare is an innovative tool for telemonitoring children with medical complexity.

In conclusion, based on our experience, the use of the Tytocare device has proven to be an effective method for the remote management of medically complex patients.

## Data Availability

The data supporting the findings of this study are available from the corresponding author upon reasonable request. All requests will be reviewed to ensure compliance with ethical guidelines and data protection policies. We are committed to providing access to the data where appropriate, in line with the principles of transparency and collaboration in scientific research.
